# Flexible outlier detection in multicenter clinical trials

**DOI:** 10.1017/cts.2026.10765

**Published:** 2026-06-05

**Authors:** Joseph Rigdon, Santiago Saldana, Sawyer Welden, W. Jack Rejeski, Edward Melanson, Neil Johannsen, Cynthia Stowe, Michael Miller

**Affiliations:** 1 Wake Forest University School of Medicine, Winston-Salem, NC, USA; 2 Noregon Systems, Greensboro, NC, USA; 3 Wake Forest University, Winston-Salem, NC, USA; 4 University of Colorado Anschutz Medical Campus, Aurora, CO, USA; 5 Louisiana State University Pennington Biomedical Research Center, Baton Rouge, LA, USA

**Keywords:** Quality control, machine learning, data repository, physical activity, open-source Software

## Abstract

**Introduction:**

Multicenter clinical trials have become increasingly larger and more complex yet assuring high-quality data remains an essential task for data coordinating centers.

**Methods:**

A flexible algorithm is presented to exhaustively search for potential data anomalies by participant and site. The algorithm proceeds as a three-phase collaboration between the data coordinating center and clinical sites. First, participant-level data are examined in a univariate approach for all relevant variables. Values at the extreme tails of the distribution that also lie outside range checks and are previously unverified by clinical sites are deemed potential outliers. Second, participant-level data are examined in a multivariate machine learning approach among meaningful groups of related variables, *e.g.,* weight and body mass index. Third, site-level differences are characterized using statistical tests and standardized differences, both adjusted and unadjusted for site demographics. Findings are discussed with sites and, if needed, alterations can be made to procedures for data collection. For illustration, the algorithm is applied to data collected in the Molecular Transducers of Physical Activity Consortium (MoTrPAC) study.

**Results:**

Application of the algorithm to MoTrPAC yielded an evaluation of over 1.9 million observations in *n* = 1029 study participants. Numerous individual univariate, multivariate, and site-level outliers were identified that were previously unidentified by existing data evaluation processes.

**Conclusion:**

It is recommended to apply this algorithm to a subset of participants early in a study, with repeated explorations over subsequent intervals throughout the study. The goal is to maximize data quality, particularly critical to the increasing occurrence of open-source, data resources.

## Introduction

Multicenter trials often form the evidence base for national medical guidelines as they are characterized by a large representative sample and a dedicated study team able to actively collect and label data that otherwise could not be obtained solely from existing data sources, *e.g.,* through the electronic health record (EHR). Well-known multicenter trials abound in the biomedical literature and are implemented in vastly diverse settings. For example, SPRINT [1] studied intensive blood pressure lowering in 102 clinics in the United States, ALMANAC [2] tested sentinel node biopsy treatment in 14 hospitals in the United Kingdom, Look-AHEAD [3] examined a behavioral diet and exercise intervention in 16 sites in the United States, and CITRIS-ALI [4] studied intravenous infusion of high-dose vitamin C in seven intensive care units in the United States. Increasingly, multicenter trials include data from new sources such as the EMR or wearable devices [5]. For example, the STRIDE study [6] used the EMR to identify eligible participants to study the effects of a lifestyle intervention. With ever-expanding and more diverse sources of data, multicenter trials continue to grow in size and complexity, increasing the data cleaning burden on data coordinating centers. Adding to the challenge, as of January 25, 2023, the NIH requires researchers to prospectively plan for how scientific data will be preserved and shared through the submission of a Data Management and Sharing Plan [7].

As multicenter trials often involve thousands of variables collected across multiple clinical sites, there is an increasing probability of data collection and data entry errors. Though few investigators report results from on-site audits [8], Neaton et al. reported error rates of 3.2 per 10,000 characters keyed for central keying, verification, and subsequent editing; 12.0 for remote keying with no verification, and 4.5 for central keying on a microcomputer with extensive error checking [9]. Because multicenter trials involve the collection of data from multiple clinical sites with diverse populations, there is some level of expected participant-level and site-level variability in the collected data. For behavioral intervention studies, participants recruited at one site may have poorer health status than participants recruited at another site due to sociodemographic and lifestyle-related factors. While this difference may vary by some reasonable amount such as 0.5 standard deviations, study personnel may worry that a systematic site-level deviation in data collection is present if the average of some measurement from participants at one site were three standard deviations greater than another site. Similarly, a participant-level outlier may be flagged if one person is three standard deviations higher or lower than the respective site-level or study-level average.

There are established best practices for collecting high-quality data in multicenter trials [10]. At the onset of a multicenter trial, a study protocol and manual of procedures (MOP) are agreed upon by site leadership to standardize data collection processes across sites. Case report forms (CRFs) are developed for groups of related variables, such as demographic characteristics, that are often collected and entered at the same time. As an example, a CRF for demographics may contain all demographic data, *e.g.,* age, sex, height, and weight, collected at baseline for all participants. Typically, the design of data entry systems for CRFs includes some level of quality assurance procedures such as the implementation of range checks, or thresholds for a variable that, if violated, would trigger investigation by study staff. For example, a BMI of 300 kg/m^2^ is obviously implausible and would be identified by a range check. Although careful planning is involved in the development of CRFs and range checks, a study can”t anticipate all possible factors that may affect data quality.

Data collection or entry errors may occur systematically at the site level due to differences in staff, equipment, machines, and technology, or simple human error. Regular quality control (QC) is another best practice for ensuring high-quality data. QC involves manual review of related groups of data points and is often divided amongst study personnel. Given the presence of thousands of variables, it quickly becomes impractical to manually QC every data point for each participant or numerous opportunities for site deviations.

There exist approaches to systematically detect outliers in multicenter trials. Trotta et al. present three unsupervised statistical monitoring algorithms [11] for detecting site-level anomalies: one using multiplicity-adjusted *p*-values from univariate participant-level statistical tests, one using a composite score of all *p*-values from a site (the Data Inconsistency Score), and one using both methods. All three methods had specificity greater than 93% in all simulated scenarios, but the method incorporating both multiplicity-adjusted p-values and the Data Inconsistency Score yielded higher sensitivity at the cost of slightly lower specificity. Sunderland et al. [12] showed that multivariate outlier detection, or considering two or more variables simultaneously, outperformed univariate outlier detection in neuropsychology and gait data from the Ontario Neurodegenerative Research Initiative. Berkowitz et al [13] used transportability methods, examining observed versus expected values of variables at sites, to identify potential site anomalies in the TOPCAT [14] (Treatment of Preserved Cardiac Function Heart Failure With an Aldosterone Antagonist) and ACCORD [15] (Action to Control Cardiovascular Risk in Diabetes–Blood Pressure) multicenter trials.

This paper goes beyond these individual contributions to provide a multi-phase approach that combines bulk univariate statistical testing, multivariate outlier detection using machine learning [16], and site-level outlier detection using adjusted and unadjusted statistical tests and standardized differences [17] to identify participant and site-level outliers. The method is illustrated via application to the Molecular Transducers of Physical Activity Consortium (MoTrPAC) [18] Study, a 10-site multicenter randomized trial collecting physiological and molecular data in response to exercise. Open-source software is provided to implement the approach.

## Materials and methods

### Algorithm for outlier detection

**Figure 1.**
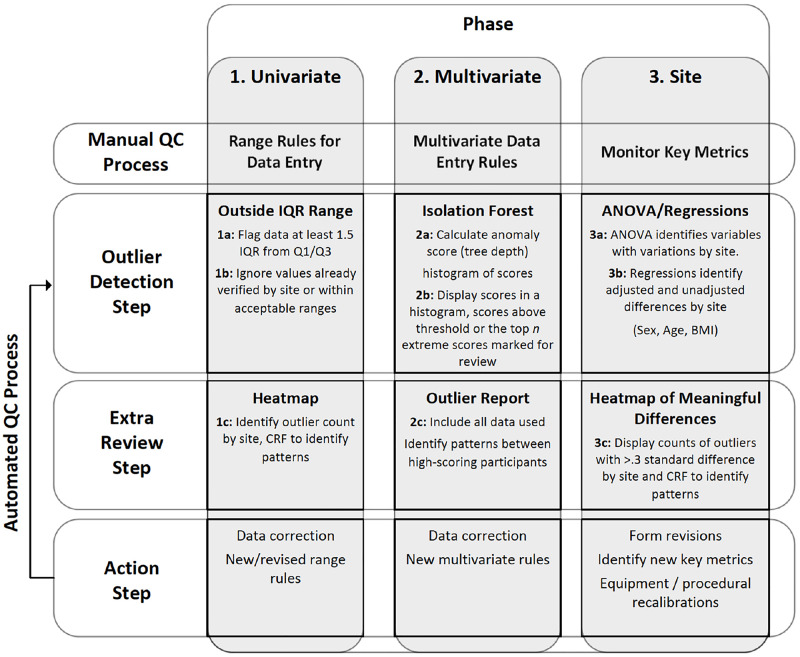
Figure 1 long description. Process map for outlier detection algorithm. The figure identifies outlier category as column headers for the three phases of data inspection. Each phase has multiple steps, which are divided into rows.

This section details of the outlier detection algorithm, also displayed in Figure 1. The three phases, each with multiple steps, are (1) univariate, (2) multivariate, and (3) site-level. Though this algorithm can flexibly accommodate user-defined groups of variables, henceforth CRFs are used to define groups of variables.

1. **Univariate Testing**
  - Box plots are created for each continuous variable. Let IQR be short for the interquartile range, Q1 for the first quartile, and Q3 for the third quartile. Following standard statistical practice [19–21], if an observation is less than Q1−1.5*IQR or greater than Q3 + 1.5*IQR, it is flagged as a potential outlier. Of note, the value of 1.5 is modifiable by users of the algorithm, *e.g*., to a more extreme value such as 3 [19].
  - Observations meeting the criteria in step 1a are cross-checked against (i) range checks, and (ii) previous verifications by study staff. If the observation is outside of range and hasn”t been previously verified, it remains a potential outlier.
  - Observations identified in steps 1a and 1b are visualized for further interpretation. Heatmaps, *e.g.,* with the *x*-axis for the site and the *y*-axis for CRF, are well-suited for this task. Along with the heatmap, it is suggested to produce a sortable listing (*e.g.,* Excel spreadsheet) that contains detailed information about each observation that has been flagged as a potential outlier. Both documents are discussed by Data Coordinating Center staff and QC working groups, as appropriate. Sites are notified via an online query tool (that integrates box plot outliers and range checks) and asked to investigate potential outliers.
2. **Multivariate outliers**. To identify outliers that may have been missed in phase 1, a multivariate approach using machine learning is employed in phase 2.
  - For each user-defined group of related variables (weight, heart rate, or other groups of important variables identified by QC working groups), isolation forests [16] are applied for multivariate outlier detection. Isolation forests proceed by building decision trees that partition each multivariate observation into its own node. Observations that are more easily partitioned, *i.e.,* have shallower average tree depth, are flagged as potential outliers. Average tree depths are mapped to a 0–1 score, where 1 indicates shallower depth. See the Appendix for complete details.
  - For each user-defined group of variables, the anomaly scores in step 2a are visualized online via histogram or other methods. Observations above a threshold (default 0.7) or with the highest-ranked anomaly scores are flagged for further review by study staff.
  - For each flagged observation, a report is generated including every variable used in the isolation forest. If several multivariate outliers are identified with similar anomalies, a broader investigation may be requested.
3. **Site-level differences.** To investigate site differences, phase 3 involves testing distributions of a variable within a CRF.
  - A global hypothesis of equal means across sites is tested via one-way analysis of variance (ANOVA). If the F-test *p*-value from one-way ANOVA is less than a user-defined significance level *p*_*g*_ (default 0.001), the variable is flagged for further examination.
  - For variables identified in step 3a, unadjusted comparisons of each site vs. all other sites are performed using two-sample *t*-tests with equal variance. If that *p*-value is less than a user-defined significance level *p*_*2*_ (default 0.05), the comparison is flagged for further investigation. A standardized difference (StDiff) calculation is also performed to record effect size. StDiffs are unitless measures mapped to a common scale and are available for continuous and categorical variables [17]. Importantly, and in contrast to *p*-values, StDiffs are not sensitive to sample size [22]. Those observations with StDiffs greater than a user-defined threshold *S* (default 0.5) are flagged for further evaluation. Adjusted comparisons are also performed using a linear regression model that includes the site (site of interest vs. all others) and a list of covariates accounting for geographic differences in recruitment, *e.g*., age, sex, and BMI. Adjusted StDiffs are calculated as the model coefficient for the site divided by the model estimated root mean squared error.
  - In the final step, flagged observations are displayed for further evaluation. Adjusted comparisons (*p*-values and StDiffs) are presented along with the unadjusted comparisons (*p*-values and StDiffs) for further context. Meetings are scheduled to review findings with QC working groups. DCC staff are present at these meetings to discuss operating procedures with the sites and how those might be tweaked to allow for higher-quality data collection.

The R package “bulkQC” includes all phases and steps of this algorithm, with version 1.1 available on the Comprehensive R Archive Network (CRAN) at the time of submission. The name “bulkQC” was chosen to indicate its ability to QC millions of data points, *i.e.,* big data tested in bulk. Interquartile range, *p*-value, and standardized difference thresholds are adjustable by the user of the R package. Multivariate outlier score and site-level *p*-value thresholds are set as sensible defaults but are easily amenable to changes by the user of the “bulkQC” software.

### Data application

The Molecular Transducers of Physical Activity Consortium (MoTrPAC) was created to study the effects of exercise on molecular structure (genomics, proteomics, metabolomics, transcriptomics) relevant to health outcomes in children, adults, and animals, across the lifespan, and with varying baseline levels of cardiovascular fitness [23,24]. In brief, MoTrPAC collected millions of data points through its studies of chronic and acute exercise in 820 rats, endurance exercise in 150 highly active adults (18+) and 50 highly active children and adolescents (10–17), and resistance exercise in 150 highly active adults (18+). Additionally, MoTrPAC planned to randomize 220 healthy sedentary children and adolescents to endurance training (*n* = 170) or non-exercise control (*n* = 50), and 1980 healthy sedentary adults to endurance exercise (EE: *n* = 840), resistance exercise (RE: *n* = 840), or non-exercise control (*n* = 300). These numbers reflect target enrollments that were altered by the COVID pandemic.

While numerous processes were put in place by the MoTrPAC bioinformatics core to ensure the validity of -omics data, the human adult sedentary randomized data are the focus of this paper. More specifically, this analysis concerns clinical phenotypic data from sedentary participants randomized to endurance exercise training (EE), resistance exercise training (RE), or non-exercise control (Control) groups. A list of relevant CRFs in MoTrPAC is presented in Table 1. Clinical and phenotypic data of interest appear on the CRFs for Acute Endurance Exercise Test (ACEE), Acute Resistance Exercise Test (ACRE), Cardiopulmonary Exercise Test (CPET), Endurance Exercise Tracking Log (EETL), Familiarizations for Endurance and Resistance Exercise (FAEA, FAEB, FARA, FARB, FARC), Grip Strength (GRIP), Height, Weight, Waist Circumference (HWWT), Isometric Knee Extension (ISKE), Local Lab Results (LABR), Pre-Screening Assessment (PSCA), Resting ECG (RECG), and Resistance Exercise Tracking Log (RETL). These CRFs were considered for univariate (phase 1) outlier detection, using measurements collected during Pre-Screening (PSC), Screening (SCP), Baseline (BAS), Intervention Session (IS), or Familiarizations (F1, F2, F3).

**Table 1.** Selected case report forms and times of collection in the molecular transducers of physical activity consortium (MoTrPAC) study. Number of variables abbreviated as # vars

			Visit^a^						
aaCase report form	Abbreviation	# vars	PSC	SCP	BAS	IS	F1	F2	F3
Acute endurance exercise test	ACEE	199	–	–	X	–	–	–	–
Acute resistance exercise test	ACRE	71	–	–	X	–	–	–	–
Cardiopulmonary exercise test (CPET)	CPET	107	–	X	–	–	–	–	–
Endurance exercise tracking log	EETL	53	–	–	–	X	–	–	–
Endurance familiarization – Session 1	FAEA	64	–	–	–	–	X	–	–
Endurance familiarization – Session 2	FAEB	62	–	–	–	–	–	X	–
Resistance familiarization – Session 1	FARA	44	–	–	–	–	X	–	–
Resistance familiarization – Session 2	FARB	43	–	–	–	–	–	X	–
Resistance familiarization – Session 3	FARC	52	–	–	–		–	–	X
Grip strength	GRIP	3		X	–	–	–	–	–
Height, weight, waist circumference	HWWT	9	–	X	–	–	–	–	–
Isometric knee extension	ISKE	6	–	X	–	–	–	–	–
Local lab results	LABR	36	–	X	–	–	–	–	–
Pre-screening assessment	PSCA	4	X	–	–	–	–	–	–
Resting ECG	RECG	1	–	X	–	–	–	–	–
Resistance exercise tracking log	RETL	86	–	–	–	X	–	–	–

a PSC = Pre-Screening, SCP = Screening, BAS = Baseline, IS = Intervention Session, F1 = Familiarization 1, F2 = Familiarization 2, F3 = Familiarization 3.

Multivariate outlier detection (phase 2) focused on groups of variables collected during the acute endurance exercise test (ACEE). External work (measured in watts), heart rate, and oxygen consumption are evaluated at minutes 14–17 and 34–37 of the ACEE. Observations with isolation forest outlier scores greater than 0.7 were flagged for further investigation.

Site-level outlier detection (phase 3) was carried out by applying steps 3a–3c of the algorithm to the variables analyzed in phase 2. The *p*-value threshold for omnibus testing was set to *p*_*g*_ = 0.001, the *p*-value threshold for (adjusted and unadjusted) site-level testing (step 3c) was set to *p*_2_ = 0.05, and the threshold for (adjusted and unadjusted) standardized differences was set to *S* = 0.5. Models included adjustment for age, sex, and BMI.

## Results

Application of the QC algorithm to the MoTrPAC phenotypic data resulted in the analysis of 840 variables on approximately the first 1,000 participants randomized. There were 10 study sites included in the analysis, spread geographically across the United States. Study sites are de-identified and labeled A–J for illustrative purposes.

**Figure 2.**
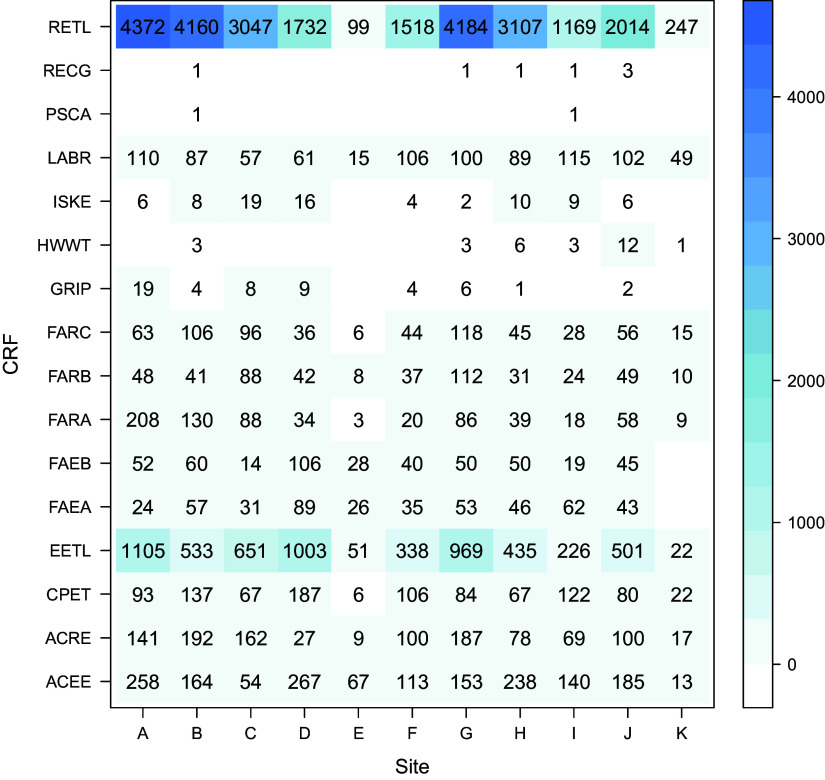
Figure 2 long description. Heatmap displaying individual univariate outliers found using interquartile range criteria alone.

**Figure 3.**
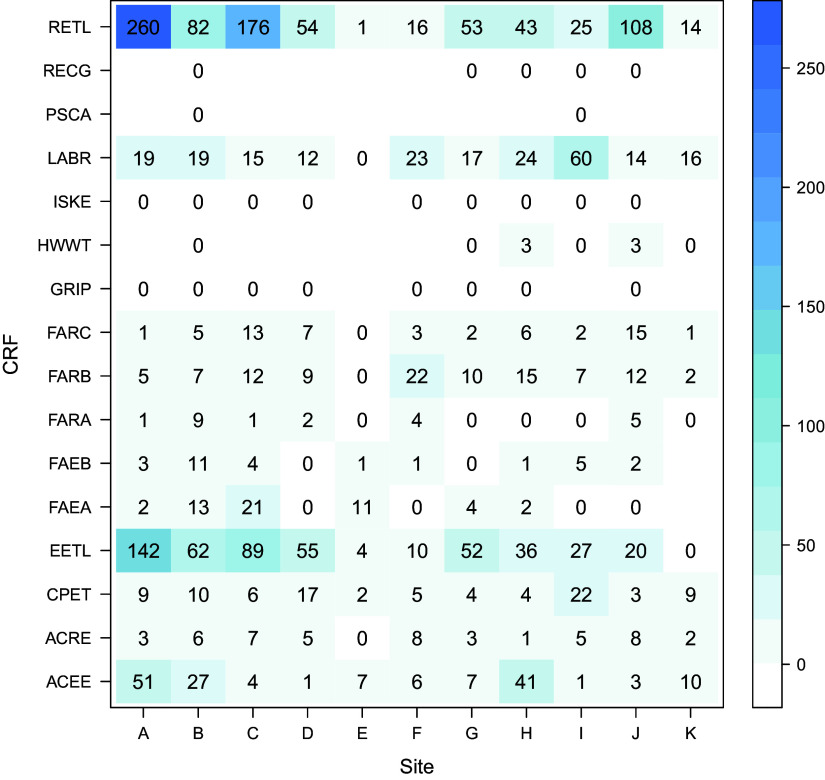
Heatmap displaying individual univariate outliers found using interquartile range criteria, and range rule checks.

**Figure 4.**
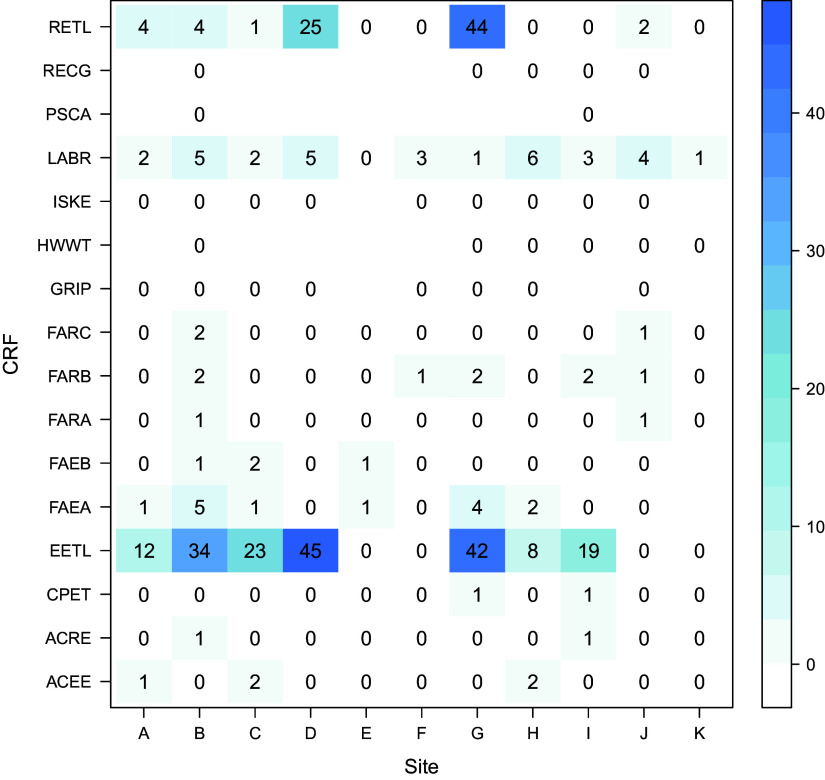
Figure 4 long description. Heatmap displaying individual univariate outliers found using interquartile range criteria, range rule checks, and query verifications.

In total, 1,920,209 observations were evaluated in Phase 1. The RETL case report form showed the most data-driven outliers after executing step 1a (Figure 2). For example, sites A, B, and G all showed over 4000 observations that were outside the whiskers on a standard boxplot. After the application of the range checks, the number of remaining outliers declined substantially (Figure 3). Whereas site A initially had 4372 outliers identified using interquartile ranges, that number is reduced to 260 after applying range checks. Numerous site-CRF combinations no longer showed any outliers after additionally applying query verifications in step 1b (Figure 4). RETL and EETL remained the most anomalous CRFs, with 6/10 sites showing at least one outlier for RETL and 7/10 for EETL. No outliers were found on GRIP, HWWT, ISKE, PSCA, and RECG. In total, 332 observations were identified for further investigation after application of Phase 1.

**Figure 5.**
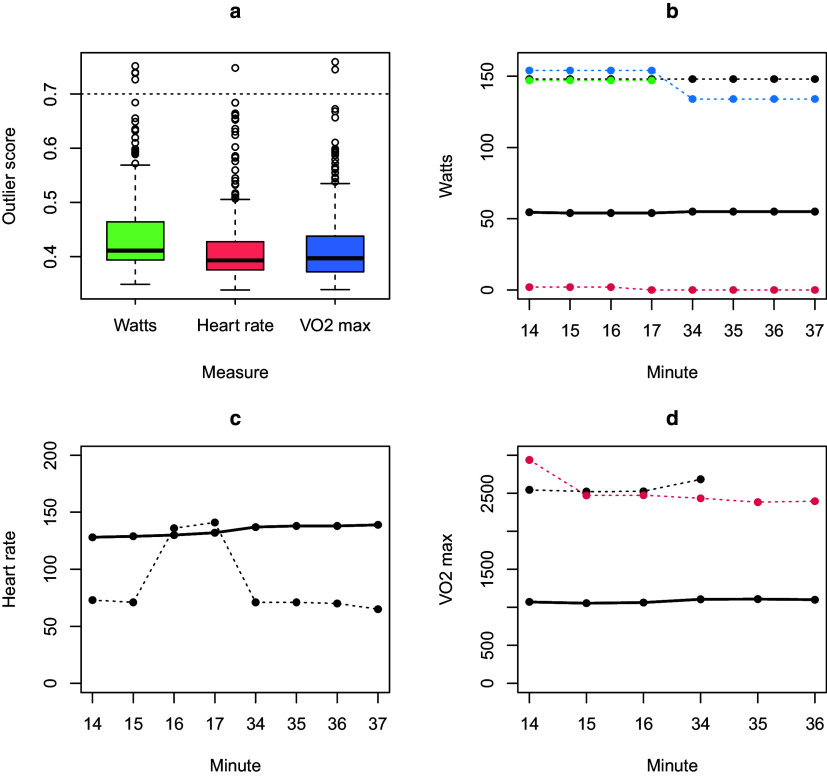
Results of application of multivariate outlier detection procedure to key measures (watts, heart rate, VO2 max) in acute endurance exercise test. Panel (a) shows boxplots of the outlier scores for all endurance exercise study participants, and panels (b–d) show trajectories of outlier observations (>0.7). In panels (b–d), the median trajectory is displayed in bold black.

Once individual outliers are identified and corrected in Phase 1, Phase 2 allows the investigation of a series of highly related measurements. Phase 2 analyses for this example concerned subsets of variables representing exertion on the acute endurance exercise test (ACEE). As displayed in Figure 5, there were a handful of the *n* = 384 endurance exercise participants to date who qualified as multivariate outliers (score >0.7) concerning their patterns of watts, heart rate, or VO_2_ max collected during important windows of the test (14–17 minutes, 34–37 minutes). More specifically, there were four study participants identified as outliers for watts, one for heart rate, and two for VO_2_ max. Figure 5b displays the trajectories of the four study participants identified as outliers for watts. Of note, one participant”s trajectory was near zero watts, whereas the median trajectory hovered around 50 W. Three participants had trajectories near 150 W. Figure 5c displays the single anomalous study participant for heart rate. The participant”s heart rate remained substantially lower than the median (75 beats per minute versus approximately 130 beats per minute) but spiked at minutes 16 and 17. Figure 5d illustrates trajectories of VO_2_ max measurements for the two study participants identified as outliers. Both participants have VO_2_ max values near 2,500, substantially greater than the median of approximately 1000.

In site-level analysis (phase 3), exertional effort (watts) showed differences between sites (Table 2). All adjusted *p*-values were less than 0.001 for watts at 14, 15, 16, 17, 34, 35, 36, and 37 minutes of the test. Unadjusted *p*-values were between 0.05 and 0.1 for all exertional effort variables. VO_2_ max values at 14, 15, and 35 minutes also showed differences between sites. Adjusted *p*-values again were all less than 0.001; however, unadjusted *p*-values were all greater than 0.05, with the largest *p*-value of 0.25 observed for VO_2_ max at 35 minutes. Table 3 displays the results of pairwise testing for the 11 (eight watts, three VO_2_ max) variables with differences between sites. Of note, site C was consistently anomalous for *p*-values and standardized differences. The single largest adjusted standardized difference (0.77) was observed for site C compared to all other sites on watts at 14 minutes. This comparison also produced the smallest adjusted *p*-value (0.0003). Site D was the only site to have unadjusted standardized differences >0.5 for any of the variables under consideration, with five such values (watts at minutes 34–37, VO_2_ max at minute 35).

**Table 2.** Subset of variables in the molecular transducers of physical activity consortium (MoTrPAC) study identified as having site-level differences

Variable	Unadjusted p-value	Adjusted *p*-value^a^
Watts, 14 minutes	0.0543	0.0001
Watts, 15 minutes	0.0617	0.0001
Watts, 16 minutes	0.0707	0.0001
Watts, 17 minutes	0.0746	0.0002
Watts, 34 minutes	0.0850	0.0002
Watts, 35 minutes	0.0811	0.0002
Watts, 36 minutes	0.0804	0.0002
Watts, 37 minutes	0.0804	0.0002
VO_2_ max, 14 minutes	0.0654	<0.0001
VO_2_ max, 15 minutes	0.0857	<0.0001
VO_2_ max, 35 minutes	0.2475	0.0004

a Adjusted for age, sex, and BMI.

**Table 3.** *P*-values and standardized differences for subset of variables in the molecular transducers of physical activity consortium (MoTrPAC) study identified as having site-level differences. Sites labeled as {A, B, …, J} to preserve de-identification. p-Values only displayed if <0.05, and standardized differences displayed if >0.5

	Watts, 14 mins	Watts, 15 mins	Watts, 16 mins	Watts, 17 mins	Watts, 34 mins	Watts, 35 mins	Watts, 36 mins	Watts, 37 mins	VO_2_, 14 mins	VO_2_, 15 mins	VO_2_, 35 mins
	*Site vs. all other sites p-values, unadjusted*										
**I**	0.0162	0.0156	0.0151	0.0152	0.0274	0.0272	0.0274	0.0274			
**C**	0.0489										
**B**									0.0394	0.0297	
	*Site vs. all other sites p-values, adjusted*										
**J**									0.0154	0.0106	0.0265
**C**	0.0003	0.0006	0.0010	0.0013	0.0016	0.0015	0.0015	0.0015	0.0278	0.0412	NA
**B**	0.0139	0.0134	0.0133	0.0132	0.0077	0.0076	0.0075	0.0075	0.0084	0.0033	0.0279
**G**	0.0423	0.0410	0.0447	0.0445							
**E**										0.0486	0.0143
	*Standardized differences, unadjusted*										
**D**					0.59	0.57	0.57	0.57			0.55
	*Standardized differences, adjusted*										
**C**	0.77	0.74	0.70	0.69	0.69	0.70	0.70	0.70			
**G**	0.69	0.69	0.68	0.68	0.52	0.52	0.55	0.55			

## Discussion

The battery of standard *manual* QC processes developed and used in MoTrPAC was extensive and included active time-intensive monitoring involving a review of key variables and range rules during data entry. In contrast to many single-site observational studies, aspects of this level of control are unique to randomized controlled trials (RCTs), wherein researchers often collect outcomes at fixed time points using highly standardized techniques across multiple sites. Planning an RCT allows researchers to consult other studies (*e.g.,* published data such as VO_2_ max in HERITAGE [25]) or conduct test–retest experiments on current instruments in advance of recruitment to understand biological vs. operator variability. Range rules for active monitoring can in turn be constructed to reflect anticipated biological variability. However, active monitoring can only be applied to a limited number of variables due to time constraints and remaining uncertainty about variability. The *automatable* QC algorithm implemented in this paper identified many potential data issues that could not have been identified using existing processes. Rather than plotting and individually inspecting thousands of trajectories, isolation forests (phase 2) were successfully applied to identify anomalous trajectories of related study variables. As this comprehensive approach was applied mid-study, it could inform the development of improved range rules and verifications for the next phase of study data collection, preventing future data anomalies and saving hours of study personnel time. As the multivariate outlier detection method, isolation forests, accommodates categorical variables, indicators of missingness could be used to detect irregular patterns of missing data, informing data collection efforts.

Participant-level and site-level outliers appearing in clinical and phenotypic data could indicate that at certain sites, human judgment may play a role in how data are being collected using different assessment procedures. Outliers in clinical data could indicate that a machine, *e.g.,* dual X-ray absorptiometry, was incorrectly calibrated or malfunctioning at a certain site. Early detection of these site-level differences, *e.g.,* via standardized differences robust to small sample sizes, can allow study personnel to intervene quickly to remedy the situation. On the other hand, some outliers may reflect true biological diversity, reflective of potential underlying genetic variations of study participants from that area. The inclusion of individuals with expertise in studying the given variables in different populations in discussions of outliers with clinical site leaders may help to differentiate data entry errors from true biological outliers.

When issues arise for specific sites, *e.g.,* for site C mentioned in the Results section, a multi-pronged approach is used to investigate. This approach involves study leadership consulting with investigators from the site to explain the results and to help them explore what issues may be leading to the anomalies. These conversations may lead to modifications of range checks, or modifications of the application of the algorithm. For example, it may be determined that 1.5 is too low of a threshold in step 1a, and a larger threshold, *e.g.,* 3 [26], may be more appropriate for outlier identification. While the application of this algorithm to MoTrPAC was influenced by existing range checks, if a study does not already have QC processes in place, 1.5 may be an appropriate choice to identify a broader range of potential outliers.

A key limitation of this study is a theme found in all automated QC procedures. When implementing automated procedures, a study team must consider the implications of choices of outlier discovery parameters (*e.g.,* the IQR in step 1a, the ranked outlier scores in 2b, and the *p*-value thresholds in 3a, 3b, and 3c). There is a trade-off between staff time for investigating anomalies and statistical rigor. Setting these discovery parameters at levels that are too sensitive may yield too much data to review for busy study staff. On the other hand, allowing these parameters to be less sensitive may miss important discoveries. The analysis presented in this paper opted for trade-offs in choosing parameters for the algorithm that would balance statistical rigor and practical considerations. Users of this procedure may want to tune those parameters to their study”s particular needs.

Numerous future directions exist. While the multivariate outlier detection method accommodates continuous and categorical data, the next implementation of the site-level outlier detection algorithm could include categorical variables [27] or variables with repeated measures [28]. A comprehensive evaluation of the current method versus other methods (such as Neaton [9], Trotta [11], and Berkowitz [13]) in simulated scenarios where the truth is known would further elucidate operating characteristics, *e.g.,* sensitivity and specificity, of the various approaches. Additional graphical displays [29] may further improve the presentation of the findings. This procedure could also be adapted to studies that incorporate data from the electronic medical record, where QC procedures may not be as standardized and rigorous across sites. Finally, advancements in computational speed could be pursued by exploiting existing approaches in R [30, 31] or considering other programming languages such as Python [32] or Julia [33]. Employing more computationally efficient approaches would make the algorithm extensible to much larger data sets, *e.g.,* -omics. In all current and future implementations of this process, iterative communication between analysts, Data Coordinating Center staff, QC working groups, and clinical site staff is paramount to address potential outliers.

## Conclusions

In summary, an algorithm was developed to aid the QC of multicenter trials with millions of observations, increasingly prevalent in the age of Big Data [34]. The algorithm was applied to phenotypic data in MoTrPAC, a 10-site multicenter trial of exercise interventions in animals, highly active and sedentary children, and highly active and sedentary adults. Participant and site-level outliers were identified using univariate, multivariate, and site-level outlier detection strategies. The algorithm is available as an open source in the R package “bulkQC,” hosted on the Comprehensive R Archive Network (CRAN).

## Supporting information

10.1017/cts.2026.10765.sm001 Rigdon et al. supplementary material Rigdon et al. supplementary material

## Data Availability

The datasets generated during and/or analyzed during the current study are not publicly available due to embargo in the MoTrPAC study but will eventually be available as part of public release datasets issued by the MoTrPAC Consortium. The functions used to analyze the data sets are available through the open-source R package “bulkQC,” hosted on the Comprehensive R Archive Network.

## Supplementary material

The supplementary material for this article can be found at https://doi.org/10.1017/cts.2026.10765.

## Figure 1. Long description

The table is divided into three main phases: Univariate, Multivariate, and Site. Each phase includes steps for Manual QC Process, Outlier Detection Step, Extra Review Step, and Action Step. The table has four rows and three columns. Column headers are Manual QC Process, Outlier Detection Step, Extra Review Step, and Action Step. Row labels are Univariate, Multivariate, and Site. Row 1: Univariate, Range Rules for Data Entry, Outside IQR Range, Heatmap, Data correction, New/revised range rules. Row 2: Multivariate, Multivariate Data Entry Rules, Isolation Forest, Outlier Report, Data correction, New multivariate rules. Row 3: Site, Monitor Key Metrics, ANOVA/Regressions, Heatmap of Meaningful Differences, Form revisions, Identify new key metrics, Equipment/procedural recalibrations.

Navigate back to Figure 1..

## Figure 2. Long description

A heat map displays values across different sites and groups, with varying color intensities indicating the magnitude of each value. The heat map is structured as a grid with rows labeled as groups and columns labeled as sites. The color scale ranges from light blue to dark blue, with darker colors representing higher values. The values range from 0 to over 4000. Notable high values are observed in the RETL group across multiple sites, particularly at sites A, F, and G. The EETL group also shows significant values at sites A and C. Other groups such as LABR, FARC, and FARB exhibit moderate values across various sites. The heat map highlights the distribution and intensity of values, indicating patterns and outliers within the data.

Navigate back to Figure 2..

## Figure 4. Long description

A heat map representing the distribution of values across different sites and groups. The heat map is structured as a grid with rows labeled as groups and columns labeled as sites. The x-axis represents different sites labeled from A to K, and the y-axis represents different groups labeled as RETL, RECG, PSCA, LABR, ISKE, HWWT, GRIP, FARC, FARB, FARA, FAEB, FAEA, EETL, CPET, ACRE, and ACEE. The color scale on the right indicates the magnitude of values, with lighter blue representing higher values and darker blue representing lower values. The range of values shown is from 0 to 45. Standout regions include high values in the EETL group at sites B, C, and G, and in the RETL group at site G. Overall, the heat map shows a concentration of higher values in specific groups and sites, with notable outliers in the EETL and RETL groups.

Navigate back to Figure 4..
