# Supplementary material for: Flexible outlier detection in multicenter clinical trials
Source: J Clin Transl Sci. 2026 Jun 5;10(1):e104. doi: 10.1017/cts.2026.10765 (PMC13323618; doi:10.1017/cts.2026.10765)
Supplement: Rigdon et al. supplementary material [file S2059866126107651sup001.docx]

**Supplementary Appendix**

**Additional Details on Isolation Forest Algorithm**

Isolation Forests^1^ detect anomalies by measuring the isolation of individual data points from the rest of the data points. Each tree in the forest consists of internal decision nodes and leaf nodes. Leaf nodes represent individual data points that have been isolated from the rest of the data by the decision nodes above them in the tree. The decision nodes have two child nodes and store a (variable, value) pair. When evaluating a decision node for a data point, if the value of the variable specified in the decision node is less than the value stored in the data point, evaluation continues with the left child node; otherwise, it continues with the right child node. Trees are trained by recursively splitting the data set with a randomly chosen variable and a value chosen uniformly at random between the minimum and maximum value for that variable in the data set. Recursion halts when a node contains a singular data point or when all remaining data points have the same values. Data points isolated using fewer decision nodes, those belonging to leaf nodes at relatively low tree depths, are thought to be more likely to be anomalous, whereas those isolated using more decision nodes and having a greater tree depth are thought to be less likely to be anomalous.

Trees in the Isolation Forest contain a random subset of data points from the data set to reduce computational load and to avoid a phenomenon called "swamping," in which an outlier is close to a non-anomalous data point in many dimensions, requiring many decision nodes on average to separate them. Under the original Isolation Forest model^1^, data points are assigned a normalized anomaly score proportional to their average tree depth and the cardinality of the dataset. The specific version used in this analysis^2^ assigns an anomaly score using an adjusted depth that considers the proportion of the dataset split onto each side of a decision node. More formally, adjusted depth d is given as $d=2/(1+\frac{1}{2p})$, with $p=(\frac{n_{s}}{n_{t}})/(\frac{r_{s}}{r_{t}})$, where $n_{s}$

is the number of points to reach a given node, $n_{t}$ is the total number of data points, $r_{s}$ is the range of the variable being split on among the points at a given node, and $r_{t}$ is the total range of the variable among all data points in the given tree.

1. Liu FT, Ting KM, Zhou ZH. Isolation Forest. In: 2008 Eighth IEEE International Conference on Data Mining. 2008:413-422. doi:10.1109/ICDM.2008.17

2. Cortes D, library) TGP (Copyright holder of included robinmap, code) DB (Copyright holder of original xoshiro, code) SV (Copyright holder of original xoshiro, tables) ND (Copyright holder of formatted ziggurat. isotree: Isolation-Based Outlier Detection. Published online September 7, 2022. Accessed December 21, 2022. https://CRAN.R-project.org/package=isotree
